# Alternatives to intravenous immunoglobulin treatment in chronic inflammatory demyelinating polyradiculoneuropathy

**DOI:** 10.1007/s00415-019-09485-9

**Published:** 2019-08-01

**Authors:** Dan Castle, Neil P. Robertson

**Affiliations:** Institute of Psychological Medicine and Clinical Neuroscience, Cardiff University, University Hospital of Wales, Heath Park, Cardiff, C14 4XN UK

## Introduction

Chronic inflammatory demyelinating polyradiculoneuropathy (CIDP) is a progressive or relapsing and remitting disease of the peripheral nervous system. It is generally thought to have an autoimmune aetiology and has a global prevalence estimated at 0.8–8.9 per 100,000. In its classical form, it is characterised by a symmetric proximal and distal weakness and sensory dysfunction of all extremities, developing over at least 2 months. Less commonly, CIDP may present with cranial nerve involvement, pure motor, pure sensory, asymmetric or predominately distal limb dysfunction.

The optimum treatment for CIDP has long been an area of debate, although with a consensus for the use of intravenous immunoglobulin (IVIg) as an induction and maintenance treatment. Trials have demonstrated that IVIg is effective in the short- and long-term treatment and well tolerated. However, there are considerable cost implications for the long-term use of IVIg, as well as the infrastructure required to administer an infusion for 2–3 days at intervals that may be as short as every 3–6 weeks. Corticosteroids, although known since 1958 to be an effective treatment choice in CIDP with response rates reported between 30 and 90%, have well-recognised cumulative side effects. However, alternative immunosuppressants such as azathioprine, methotrexate or rituximab are commonly viewed as alternatives to IVIg particularly in healthcare systems that may be unable to provide invasive treatments such as plasma exchange or IVIg. As a result, research has focussed on identifying alternative immunomodulating agents that are more convenient, cost effective and improve patient outcomes and autonomy.

This month’s journal club will review three treatment options in the management of CIDP. The first paper reviews the use of subcutaneous immunoglobulin (SCIg) in the PATH trial; the second looks at the use of different regimes of corticosteroids and the last reviews the novel use of fingolimod in the FORCIDP trial.

## Subcutaneous immunoglobulin for maintenance treatment in chronic inflammatory demyelinating polyneuropathy (PATH)

This international, randomised, double-blind, placebo-controlled, phase 3 trial reviewed the use of two different doses of SCIg in CIDP. SCIg has been already been used for many years in conditions such as primary immunodeficiency, with proven efficacy and is often preferred by patients. In this study, 172 patients received either SCIG at a dose of 0.4 g/kg (high dose), 0.2 g/kg (low dose) or placebo. Following screening, dependency on IVIg was assessed by withholding current IVIg treatment for 12 weeks and monitoring response. Those deemed dependent were then put through an IVIg re-stabilisation period to acquire a standardised regime. Patients were assessed using the Inflammatory Neuropathy Cause and Treatment (INCAT) scoring system at the end of the re-stabilisation period, which acted as baseline for future assessments. Those successfully re-stabilised were then entered into a 24-week treatment period.

Primary outcome measure was the proportion of patients who had a relapse or were withdrawn from the study for any reason during the treatment period. The absolute risk reduction for the primary outcome was 25% in the low-dose group and 30% in the high-dose group compared with placebo. The probability of remaining relapse free by the end of the treatment period was estimated to 77.6% in the high-dose group, 65.0% in the low-dose group and 41.2% in the placebo group. The number needed to treat (NNT) to prevent one relapse of CIDP was 2.7 in the high-dose group and 4.4 in the low-dose group. SCIg was well tolerated by patients, with adverse event rates equal across all three groups with the majority for minor skin reactions. Patients also reported a preference for the use of SCIg, as it improved autonomy and had fewer side effects.

*Comment* This well-designed study demonstrates comparable NNT (2.7, 4.4) to IVIg in CIDP as calculated in a Cochrane review (3.03). It also highlights some positive patient outcomes for a treatment that can be lifelong. Limitations of the study included patient numbers despite the involvement of 69 neuromuscular centres. In addition, although attempts were made to ensure patients were IVIg dependent, 44% of patients in the placebo group did not relapse. Whilst this phenomenon is common in CIDP trials, it does demonstrate some of the practical difficulties of identifying appropriate cohorts despite wide engagement by relevant centres.

van Schaik et al. (2018) The Lancet Neurology; 17: 35–46.

## Corticosteroids in chronic inflammatory demyelinating polyneuropathy

There is currently no consensus for the use of a common corticosteroid regime in CIDP. In this multinational retrospective analysis, treatment naïve CIDP patients prescribed a defined regime of oral prednisolone, pulsed oral dexamethasone or intravenous methylprednisolone were reviewed.

Patients who met the European Federation of Neurological Societies/Peripheral Nerve Society diagnostic criteria for (EFNS/PNS) for definite, probable, or possible CIDP were included. Those deemed to have severe, ‘fast progressive’ or a pure motor phenotype CIDP were excluded. Disease severity at baseline was assessed by the medical research councils (MRC) sum score and a modified Rankin scale score (mRS). Primary outcome was the number of responders in each arm, defined as any patient who showed improvement in motor or sensory impairment as captured by the treating neurologist and/or the mRS, and did not require additional treatment for CIDP. Primary outcome was assessed at 6 months from treatment onset. Secondary outcomes included, time to relapse, adverse events and 5-year remission rate as measured on the CIDP disease activity status score (CDAS).

In total, 125 patients were recruited, 58 from Serbia with 98% of these taking the defined prednisolone regime. From the Netherlands, 43 patients were recruited with 86% taking the dexamethasone regime. Finally, 24 patients were recruited from Italy with 83% taking the methylprednisolone regime. Overall, 75 (60%) of patients met the primary outcome measures and were considered responders. There was no statistical significance between the three groups for the primary outcome. Of the 75 responders, 46 (61%) remained remission free during a median follow-up of 55 months (1–197) and 55% of the patients achieved a 5-year remission. For all 125 patients, the 5-year remission rate was 33%.

*Comment* This study explored three corticosteroid regimes, with a long follow-up period. Limitations include uneven distribution of patients between the regimes (54% of patients were in the prednisolone arm of the study) and the potential for inter-observer bias. Although there was no statistical significance between the three groups, pulsed dexamethasone provided the smallest cumulative dose of corticosteroid and 91% of patients responded to either corticosteroids alone, or corticosteroids followed by IVIg.

van Lieverloo et al. (2018) Journal of Neurology; 265: 2052–2059.

## Oral fingolimod for chronic inflammatory demyelinating polyneuropathy (FORCIDP trial): a double-blind, multicenter, randomised control trial

Fingolimod is a sphingosine 1-phosphate receptor modulator with a well-established role in the treatment of relapsing multiple sclerosis and causes retention of autoreactive T cells in lymph nodes with subsequent reduction in circulating naïve T and B cells. In this study by Hughes et al., patients previously on either IVIg or corticosteroids for CIDP were recruited from 14 countries and given fingolimod or placebo in a double-blind randomised trial. Patients were required to meet the EFNS/PNS definition for CIDP, have an INCAT score at screening of 1–9 and have a documented clinical deterioration in the last 18 months but should have been clinically stable in the 6 weeks prior to randomisation. Primary outcome was time to first confirmed worsening, defined by an increase in the INCAT score of at least one. An interim analysis was planned after 50 observed events.

One hundred and six patients were recruited and randomised. The study was ended at 3 years when the interim analysis concluded that there was no difference between the two groups for any of the study outcomes. Confirmed worsening was seen in 42% of the fingolimod group and 43% of the placebo group, with a hazard ratio of 1.0 calculated for the use of fingolimod.

*Comment* This study found no evidence for benefit of fingolimod in CIDP. The authors did note that the relapse-free rate in the placebo group was 42% indicating that patients with inactive CIDP were included in the study. Although some attempts were made to exclude patients with inactive disease, using a similar screening step to that used in PATH trial could have been more beneficial. In addition, patients on IVIg started treatment the day after last dose, whereas those on corticosteroids started on initiation of their dose tapering. This resulted in patients on corticosteroids having a more gentle transition to the study drug than those on IVIg. Fingolimod is estimated to take 2–6 weeks to achieve full activity and therefore this may have resulted in patients relapsing before it had reached maximal efficacy.

Hughes et al. (2018) The Lancet Neurology; 17: 689–698.

